# Sleep quality in the Croatian adult population during the COVID-19 pandemic

**DOI:** 10.1093/eurpub/ckac131.479

**Published:** 2022-10-25

**Authors:** I Miskulin, I Fotez, M Miskulin, J Milas, H Palenkic, M Kristic

**Affiliations:** 1 Department of Public Health, Faculty of Medicine Osijek, University of Osijek, Osijek, Croatia; 2 Department of Clinical Medicine, Faculty of Dental Medicine and Health Osijek, Osijek, Croatia; 3 Department of Psychiatry, University Hospital Split, Split, Croatia

## Abstract

**Background:**

The COVID-19 pandemic as a worldwide public health issue is a traumatic event that has affected both the sleep and mental health of the general population. This study aimed to evaluate the quality of sleep in the Croatian adult population during the COVID-19 pandemic.

**Methods:**

This cross-sectional questionnaire study was conducted from February to June 2021 period. A validated, anonymous questionnaire that contained questions regarding demographic data, as well as Pittsburgh Sleep Quality Index (PSQI) and Coronavirus Anxiety Scale (CAS) was self-administered to a convenient sample of Croatian adults from central and northwestern Croatia.

**Results:**

The study sample included 939 subjects with, median age of 42 years (interquartile range 35-48), 35.4% males, and 64.6% females. At the PSQI 22.6% of subjects presented sleep disturbances while at the CAS 0.4% of subjects presented dysfunctional anxiety associated with the COVID-19 pandemic. Sleep disturbances were more frequent among females (p < 0.001), inhabitants of the Croatian capital Zagreb (p = 0.001), subjects who were not infected with COVID-19 virus (p = 0.042), subjects who had fear of coronavirus infection in the workplace (p < 0.001), subjects who had fear of coronavirus infection during daily life activities (p < 0.001), subjects who had fear of coronavirus infection during daily physical activities (p < 0.001), subjects who worked with limited social contact (p = 0.005), and subjects with dysfunctional anxiety associated with the COVID-19 pandemic (p = 0.003).

**Conclusions:**

Poor sleep quality is common during the COVID-19 pandemic in Croatia. Identifying factors associated with poor sleep would help develop specific intervention programs that enhance mental health and sleep quality during pandemics.

**Key messages:**

• The COVID-19 pandemic has a significant negative influence on the mental health of the Croatian general population.

• Appropriate supportive programs and interventional approaches directed toward the general population are needed to address mental health problems in Croatia during the COVID-19 pandemic.

